# The long noncoding RNA MAPKAPK5-AS1 promotes the formation of hepatitis B virus cccDNA by modulating the expression of NABP1

**DOI:** 10.1080/21505594.2026.2745300

**Published:** 2026-10-12

**Authors:** Xiaolu Zhang, Tingting Liu, Qiuyue Jiang, Zhijie Hu, Shan Cen, Quan Zhang

**Affiliations:** a Joint Institute of Nanjing Drum Tower Hospital for Life and Health, College of Life Science, Nanjing Normal University, Jiangsu, China; b Department of Laboratory Medicine, Nanjing Drum Tower Hospital and Jiangsu Key Laboratory for Molecular Medicine, The Affiliated Hospital of Nanjing University Medical School, Jiangsu, China; c Department of Laboratory Medicine, Nanjing Drum Tower Hospital, The Affiliated Hospital of Nanjing University Medical School, Jiangsu, China; d Institute of Medicinal Biotechnology, Chinese Academy of Medical Science, Beijing, China

**Keywords:** Long noncoding RNAs, hepatitis B virus, MAPKAPK5-AS1, nucleic acid binding protein 1, covalently closed circular DNA

## Abstract

Cellular long noncoding RNAs (lncRNAs) have been shown to participate in diverse biological pathways, including the regulation of virus infections. However, their functions in the hepatitis B virus (HBV) life cycle still need to be explored. Here, we conducted a loss-of-function screen targeting 1779 human lncRNAs and identified 19 lncRNAs associated with HBV infection. One lncRNA, named MAPKAPK5-AS1 (lnc-MAAS), was identified as a host factor required for HBV infection. Our data suggest that lnc-MAAS is involved in the conversion of HBV relaxed circular DNA into covalently closed circular DNA (cccDNA). Furthermore, mechanistic experiments revealed that lnc-MAAS affects cccDNA formation by modulating the expression of nucleic acid binding protein 1 (NABP1). Together, our findings shed light on the roles of host lnc-MAAS and NABP1 in HBV cccDNA formation, which may contribute to the development of novel anti-HBV therapeutics.

## Introduction

Long noncoding RNAs (lncRNAs) are a class of transcripts that are longer than 200 nucleotides and have no apparent protein-coding capacity [1]. The human genome encodes at least 19,000 annotated lncRNA genes, divided into intergenic, exonic, intronic, or overlapping lncRNAs on the basis of their genomic position [2]. LncRNAs are expressed in a cell-type- or tissue-specific manner and are frequently dysregulated in many human diseases [3]. Although the biological functions of most lncRNAs remain elusive, it has been well proven that lncRNAs are not transcriptional “noise” but rather participate in diverse cellular processes, including the regulation of virus infections [4,5].

Hepatitis B virus (HBV), a small enveloped hepatotropic DNA virus, chronically infects approximately 254 million people worldwide [6]. The persistence of viral covalently closed circular DNA (cccDNA) is the root cause of chronic HBV infection, which constitutes a high-risk factor for progressive liver diseases, including liver cirrhosis and hepatocellular carcinoma (HCC). HBV cccDNA is located in the nuclei of infected hepatocytes and serves as the template for all viral transcription. The long-term presence of cccDNA and continuous viral replication drive hepatic cellular damage through various mechanisms. Wang et al. demonstrated that HBV promotes disease progression through dysregulated autophagy [7], while Ning et al. and Ramalingam et al. showed that specific HBV mutations are associated with increased hepatocellular carcinoma risk [8,9]. Currently, HBV cccDNA cannot be eliminated by approved anti-HBV drugs, including type I interferons and nucleos(t)ide analogs [10]. To identify novel targets for curing HBV, improving our understanding of the HBV life cycle in human hepatocytes is highly important.

The HBV genome is approximately 3.2 kb long and encodes only 7 viral proteins [11]. Thus, multiple host factors in human hepatocytes are indispensable for successful HBV infection [12]. Previous studies on HBV‒host interactions have focused mainly on investigating the functions of host protein-coding genes in the HBV life cycle. However, recent studies suggest that cellular long noncoding RNAs (lncRNAs) also play important roles in HBV replication [13]. For example, the lncRNA DLEU2 can enhance HBV replication by directly binding to the HBV X protein to sustain cccDNA transcription [14]. In contrast to DLEU2, the lncRNAs HOTTIP and LINC01431 act as host restriction factors for HBV transcription. HOTTIP suppresses HBV replication through the upregulation of HOXA13, which can bind to the HBV enhancer I and X promoters to repress the production of viral RNA [15]. The lncRNA LINC01431 can stabilize type I protein arginine methyltransferase, promoting histone H4R3 methylation of the HBV cccDNA minichromosome to restrain cccDNA transcription [16].

Thousands of annotated lncRNA genes are expressed in hepatic cells [17]. Although few lncRNAs have been shown to actively participate in HBV replication, the interactions between HBV and host lncRNAs are still largely unexplored. In the present study, to identify novel host lncRNAs involved in the HBV life cycle, we employed an endoribonuclease-prepared small interfering (esiRNA) library targeting 1779 individual human lncRNAs to perform a loss-of-function screen for HBV infection. A host lncRNA, named MAPKAPK5-AS1 (lnc-MAAS), was identified as a host factor required for HBV infection. Moreover, our data suggest that lnc-MAAS functions in viral covalently closed circular DNA (cccDNA) formation by promoting the expression of nucleic acid binding protein 1 (NABP1), a single-stranded DNA‑binding protein that participates in the cellular DNA damage response.

## Materials and methods

### Plasmids, antibodies, and reagents

The lnc-MAAS-expressing plasmid was constructed by inserting the synthesized full-length lnc-MAAS DNA sequence into the pCDH-CMV-EF1-GFP vector (System Bioscience, Palo Alto, USA). The plasmid expressing NABP1 sgRNA (targeting sequence: GTCCTCGATAATCTGGGTT) and Cas9 was constructed by inserting the synthesized targeting DNA sequence into the lentiCRISPRv2 vector. For construction of the NABP1 promoter reporter plasmid, the promoter region of the NABP1 gene (*Homo sapiens* chromosome 2, GRCh38.p14 Primary Assembly, from 191,676,141 to 191,678,146) was amplified via PCR and inserted into the pGL3-Basic vector (Promega, Madison, USA). The hepatitis B core antigen (HBcAg) antibody (Catalog number GB058629) was purchased from Gene Tech (Shanghai, China), and the NABP1 antibody (Catalog number 16,719–1-AP) was purchased from Proteintech (Rosemont, USA). The lncRNA esiRNA library containing 1779 esiRNAs (Catalog number ESILHNC-1UG) and the esiRNAs targeting lnc-MAAS (Catalog number EHNC028701) were purchased from Merck (Darmstadt, Germany). The ELISA kits for detecting hepatitis B e antigen (HBeAg) (Catalog number X20231110B) and hepatitis B surface antigen (HBsAg) (Catalog number B20240404A) were obtained from Wantai (Beijing, China). Actinomycin D (Catalog number HY-17559) was purchased from MedChemExpress LLC (Shanghai, China).

### Cell culture and transfection

The human hepatoblastoma cell line HepG2 and the HepG2-derived stable cell line HepG2.2.15 were cultured in Eagle’s minimal essential medium (EMEM) (WISENT INC., Saint-Jean-Baptiste, Canada, Catalog number 320–006-CL) supplemented with 10% fetal bovine serum (FBS) (VivaCell, Shanghai, China, Catalog number C04001) and 2 mM L-glutamine. Huh7 cells were maintained in Dulbecco’s modified Eagle’s medium (DMEM) (VivaCell, Shanghai, China, Catalog number C3113) with 10% FBS and 2 mM L-glutamine. HepG2-hNTCP cells were constructed as described previously [18] and cultured in EMEM supplemented with 10% FBS, 2 mM L-glutamine, and 1 μg/mL puromycin (Selleck Chemicals LLC, Houston, USA, Catalog number S7417). HepAD38 cells were maintained in EMEM containing 10% FBS, 2 mM L-glutamine, 1 μg/mL tetracycline (MedChemExpress LLC, Shanghai, China, Catalog number HY-A0107), and 400 μg/mL G418 (MedChemExpress LLC, Shanghai, China, Catalog number HY-17561). The primary human hepatocytes were purchased from Liver Biotechnology (Shenzhen, China) and cultured according to the manufacturer’s instructions. For esiRNA or siRNA transfection, Lipofectamine RNAiMAX reagent (Invitrogen, Carlsbad, USA, Catalog number 13,778,075) was used to reverse‑transfect the cells. For plasmid transfection, Lipofectamine 3000 reagent (Invitrogen, Carlsbad, USA, Catalog number L3000008) was used.

### Establishment of the HepG2-hNTCP NABP1 knockout cell line

The plasmid expressing Cas9 and NABP1 sgRNA was cotransfected with ViraPower Packaging Mix (Invitrogen, Carlsbad, USA, Catalog number K4975-00) into 293T cells. Two d post-transfection, the lentiviral vectors in the supernatant were harvested and used to infect HepG2-hNTCP cells. Single-cell colonies were obtained via the limited dilution method and then analyzed by Sanger sequencing and immunoblotting to identify positive NABP1 knockout clones.

### HBV preparation and infection

HepAD38 cells, which produce genotype D HBV, were cultured in the absence of tetracycline and G418 for 7 d. The culture medium was subsequently collected every 3 d for 15 d. The collected medium was spun down at 1000 × g for 5 min to remove any cell debris and then concentrated 50-fold via 8% PEG 8000. HBV genotypes A, B, and C viruses were prepared and concentrated as previously reported [19]. The concentrated virions were used to infect HepG2-hNTCP cells in the presence of 4% PEG 8000 and 2% DMSO, as described previously [19].

### esiRNA screening

HepG2-hNTCP cells were reverse-transfected in 96-well plates with 30 nM individual esiRNAs via 0.2 µL of Lipofectamine RNAiMAX per well. Two d after transfection, the cells were infected with a 50-fold concentrated HBV inoculum as described above. Twenty-four hours post-infection, the cells were washed with PBS and cultured with fresh EMEM supplemented with 10% FBS and 2% DMSO. The culture medium of the infected cells was changed every 2 d. On day 5 post‑infection, the culture supernatant of the infected cells was harvested to measure the level of HBeAg by ELISA.

### Detection of HBcAg

For detection of HBcAg, HBV-infected cells were washed and fixed in 4% paraformaldehyde, permeabilized, and stained with an HBcAg antibody (1:200 dilution) as described previously [20]. Then, the stained cells were labeled with Tyramide SuperBoost Kits (Invitrogen, Carlsbad, USA, Catalog number B40944) and visualized with a DMi8 microscope (Leica, Wetzlar, Germany).

### Isolation and detection of HBV and cellular RNAs

Total RNA was extracted from transfected or infected cells via the RNAiso Plus reagent (Takara, Shiga, Japan, Catalog number 9108Q) following the manufacturer’s instructions. The extracted RNA was subsequently converted into cDNA via the PrimeScript RT reagent Kit with gDNA Eraser (Takara, Shiga, Japan, Catalog number RR047A). HBV RNA and host lncRNAs were quantified via qPCR with the primers listed in Supplementary Table 1.

### Cell fractionation

Cell fractionation was performed via a nuclear/cytoplasmic isolation kit (Abbkine Biotechnology Co., Ltd., Wuhan, China, Catalog number KTP4002) according to the manufacturer’s instructions. RNAs in the isolated nuclear and cytoplasmic fractions of cells were extracted and converted into cDNA, as described above.

### Quantification of HBV cccDNA

For the detection of HBV cccDNA by qPCR, total cellular DNA was extracted via a TIANamp Genomic DNA Kit (TIANGEN, Beijing, China, Catalog number GDP304-02) following the manufacturer’s instructions. The extracted DNA was subsequently treated with T5 exonuclease (NEB, Ipswich, USA, Catalog number M0663S) to eliminate HBV-relaxed circular DNA (rcDNA) [21] and HBV cccDNA was quantified with the primers listed in Supplementary Table 1. For the detection of HBV cccDNA by Southern blot, protein-free forms of HBV-DNA including cccDNA were extracted using a modified Hirt extraction method [22]. Southern blot analysis was performed as previously reported [23]. Briefly, equal amounts of extracted DNA were subjected to 1.2% agarose gel electrophoresis and then transferred onto an Amersham Hybond-N+ membrane (GE Healthcare). After being fixed by UV crosslinking, the membrane was hybridized with HBV probes prepared with the random primer DNA labeling kit, ver. 2 (TaKaRa, Shiga, Japan, Catalog number 6045). Finally, the hybridization signal was visualized using a Typhoon FLA 9500 imager (GE Healthcare Lifesciences, USA).

### HBV attachment and internalization assay

Suspended HepG2-hNTCP cells were mixed with 50-fold concentrated HBV inoculum in the presence of 4% PEG 8000. After incubation at 4°C for 3 h to allow HBV to attach to the cells, free HBV was removed by washing the cells five times with PBS. To measure the amount of attached HBV, total DNA from the washed cells was extracted with a TIANamp Genomic DNA Kit (TIANGEN, Beijing, China, Catalog number GDP304-02), and HBV DNA was quantified by qPCR with the primers listed in Supplementary Table 1. To measure internalized HBV, the washed cells were cultured in a humidified 5% CO_2_ incubator at 37°C for an additional 16 h. The cells were then harvested with trypsin and washed with PBS. Total DNA was extracted from the cells, and HBV DNA was quantified as described above.

### RNA sequencing

Poly(A)-containing mRNA was enriched from 1 μg of total RNA using oligo(dT)-coated magnetic beads. The captured mRNA was then subjected to chemical fragmentation at elevated temperature (85°C for 6 min) to generate RNA fragments of approximately 280–380 nt. First-strand cDNA was synthesized using random hexamer primers and reverse transcriptase, followed by second-strand cDNA synthesis using DNA polymerase I and RNase H. The resulting double-stranded cDNA was end-repaired, A-tailed, and ligated with sequencing adapters. Adapter-ligated products were size-selected (400–500 bp) by SPRI beads and PCR amplified (11 cycles) to generate the final library. Sequencing was performed on the NovaSeq 6000 platform with paired-end reads (2 × 150 bp).

### Statistical analysis

The differences between groups were estimated by Student’s *t* test (two-tailed) or a one-way analysis of variance (ANOVA). A *p* value < 0.05 was considered significant in all tests.

## Results

### Identification of host lncRNAs associated with HBV infection

To identify novel host lncRNAs associated with HBV infection, we performed a loss-of-function screen using the HepG2-hNTCP cell model (Figure 1(A)). The reliability of the assay was verified via the use of an esiRNA against the host factor sodium taurocholate cotransporting polypeptide (NTCP), a functional receptor for HBV. As expected, the knockdown of NTCP expression significantly decreased the HBeAg level in the culture supernatant of infected cells (Figure 1(B)).

**Figure 1.**
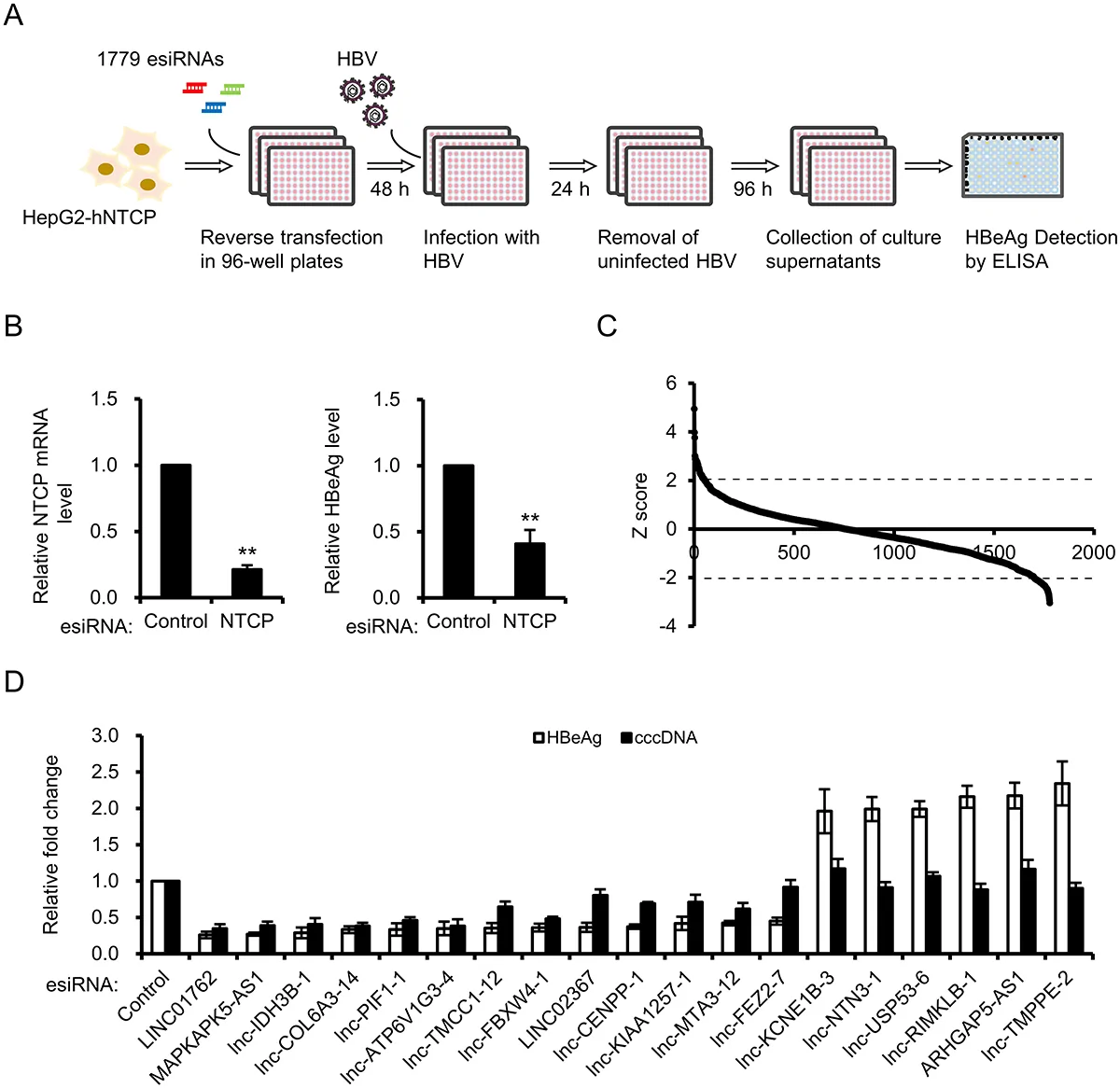
EsiRNA screen for host lncRNAs associated with HBV infection. (A) Schematic illustration of the screen. (B) Validation of the assay reliability using an esiRNA against NTCP. The expression of NTCP was measured by qPCR, and the level of HBeAg was determined by ELISA. (C) The plot of Z scores for all individual esiRNAs in the library. Each esiRNA was ranked by its Z score. Z score thresholds of −2 and 2 are shown as broken lines. (D) Effects of identified host lncRNAs on HBV infection. HepG2-hNTCP cells were transfected with the indicated esiRNAs for 48 h and infected with HBV for 24 h. Five d post-infection, HBeAg in the supernatants and cccDNA in the cells were measured via ELISA and qPCR, respectively. The results are shown as the mean values ± standard deviations. Statistical analysis was performed via Student’s *t*-test (two-tailed). *p < 0.05, **p < 0.01. HBeAg, hepatitis B e antigen; NTCP, sodium taurocholate-transporting polypeptide; cccDNA, covalently closed circular DNA.

Using this assay, we screened an esiRNA library that targets 1779 individual human lncRNAs. The primary hits were selected on the basis of a Z score ≤ −2 or a Z score ≥2 (Figure 1(C)). After excluding toxic esiRNAs and non-expressed lncRNAs, we identified 19 lncRNAs associated with HBV infection (Supplementary Table 2). Among these 19 lncRNAs, the depletion of 13 lncRNAs reduced the secreted HBeAg level, indicating that they are required for HBV infection. In contrast, knockdown of the other 6 lncRNAs significantly increased the secreted HBeAg level (Figure 1(D)). Since cccDNA is the most important marker of HBV infection, we next determined the effects of these candidates on the level of HBV cccDNA. Our data revealed that the knockdown of 13 lncRNAs that positively regulate HBV infection mostly decreased cccDNA levels (Figure 1(D)). Interestingly, the other 6 lncRNAs that negatively regulate HBV infection had no significant effect on the cccDNA level (Figure 1(D)), suggesting that they act on the transcriptional or post-transcriptional stages of the HBV life cycle.

### Characterization of lnc-MAAS

Among the 19 identified lncRNAs, MAPKAPK5-AS1 (lnc-MAAS) was selected for further characterization for several reasons. First, to date most reported lncRNAs participate in the HBV life cycle by modulating cccDNA transcription without affecting the level of cccDNA [14–16]. Therefore, we are more interested in the lncRNAs that can regulate HBV cccDNA levels. In our primary screen, lnc-MAAS knockdown significantly decreased the level of cccDNA (Figure 1(D)). Second, in the published literature lnc-MAAS can be stabilized by the HBV protein HBeAg [24], indicating that it might play a role in the HBV life cycle. Third, the expression level of lnc-MAAS in HepG2 cells is relatively higher than that of other candidates (Supplementary Table S2).

The human lnc-MAAS gene is located on the reverse strand of chromosome 12. Lnc-MAAS has multiple isoforms, and the esiRNA used in our study was designed against the common sequence stretches of lnc-MAAS (Figure 2(A)). By running the Coding Potential Calculator (CPC) program, we found that the CPC scores of all isoforms of lnc-MAAS were similar to those of the validated lncRNA NEAT1 but much lower than those of the protein-coding gene GAPDH (Figure 2(B)), suggesting that lnc-MAAS lacks coding potential. Next, we investigated the subcellular localization of lnc-MAAS via qPCR analysis of separated cytoplasmic and nuclear RNAs. The unspliced glyceraldehyde-3-phosphate dehydrogenase (GAPDH) RNA (pre-GAPDH), which is exclusively localized in the nucleus, served as a control [25]. Our data revealed that lnc-MAAS was localized in both the cytoplasm and the nucleus of HepG2 cells (Figure 2(C)). A previous study reported that the expression level of lnc-MAAS was greater in HBV-related HCC samples than in non-HBV-related HCC samples [24]. Consistently, we found that lnc-MAAS was significantly upregulated in stable HBV-expressing cell lines, including HepG2.2.15 and HepAD38 (Figure 2(D)), and in HBV-infected HepG2-hNTCP cells (Figure 2(E)).

**Figure 2.**
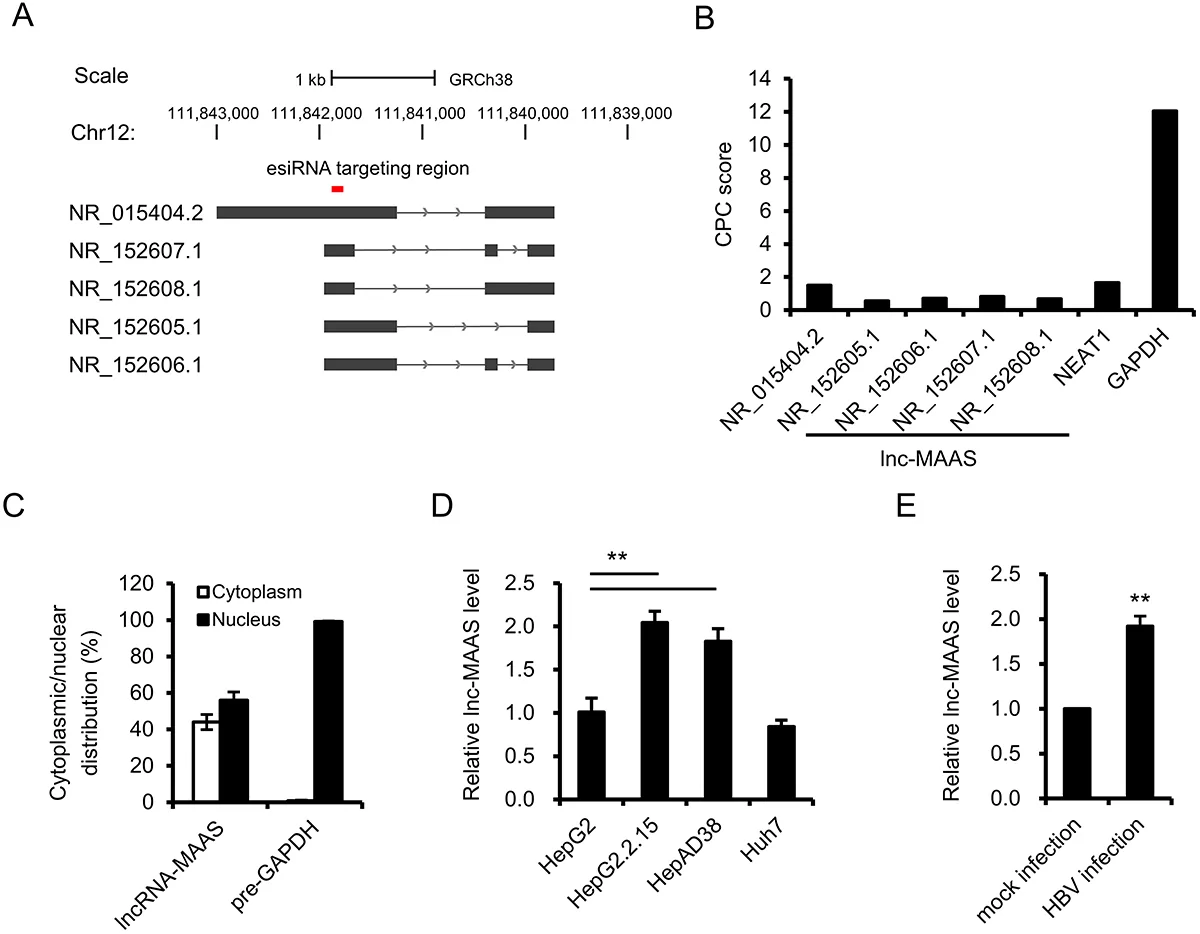
Characterization of lnc-MAAS. (A) Schematic representation of the isoforms of human lnc-MAAS. The position of the sequence targeted by lnc-MAAS esiRNA are indicated with a black box. (B) CPC scores of all isoforms of lnc-MAAS. NEAT1 and GAPDH served as controls for validated lncRNA genes and validated protein-coding genes, respectively. (C) The levels of lnc-MAAS RNA in the cytoplasmic and nuclear fractions of HepG2-hNTCP cells were analyzed by qPCR. The unspliced GAPDH RNA (pre-GAPDH), which is exclusively localized in the nucleus, served as a control. (D) The expression levels of lnc-MAAS in different hepatoma cells were determined by qPCR. GAPDH was used as the internal control. The level of lnc-MAAS in HepG2 cells was set as 1. (E) HepG2-hNTCP cells were infected with concentrated HBV inoculum at an MOI of 2000 or with the same volume of concentrated culture medium. Ten d post-infection, the infected cells were harvested, and the expression level of lnc-MAAS was determined via qPCR. The results are shown as the mean values ± standard deviations. Statistical analysis was performed via two-tailed Student’s *t*-test (E) or one-way ANOVA with Dunnett’s posttest (D). *p < 0.05. CPC, coding potential calculator.

### Lnc-MAAS is required for HBV infection

To validate the effect of lnc-MAAS on HBV infection, we transfected lnc-MAAS esiRNA into HepG2-hNTCP cells and then infected the cells with HBV. Our data showed that downregulation of lnc-MAAS dramatically reduced both secreted HBeAg and HBsAg levels by approximately 70% (Figure 3(A,B)). In addition, we stained the infected cells for HBcAg and found that the results were consistent with the ELISA data (Figure 3(C)). Next, we measured the effect of lnc-MAAS depletion on the HBV cccDNA level via qPCR and Southern blot, respectively. The results showed that the knockdown of lnc-MAAS can reduce cccDNA levels by approximately 60% (Figure 3(D)). Notably, the effect of lnc-MAAS knockdown on HBV infection was observed across all tested HBV genotypes (A, B, C, and D), suggesting that lnc-MAAS exerts a pan-genotypic proviral function (Supplementary Figure S2). To exclude the possibility that the inhibition of HBV infection by lnc-MAAS esiRNA was a result of cell toxicity, we performed both CCK-8 and Annexin V/PI assays. The results revealed that lncRNA-MAAS silencing by esiRNA had no significant effect on cell viability or apoptosis (Figure 3(E,F)). Furthermore, to confirm our data, we also employed chemically synthesized siRNA to silence lnc-MAAS, which provided results consistent with those from esiRNA-mediated knockdown of lnc-MAAS (Supplementary Figure S1). Next, we investigated the effect of lnc-MAAS overexpression on HBV infection. The results revealed that overexpression of lnc-MAAS moderately but significantly increased HBV infection by approximately 30% (Figure 3(G)).

**Figure 3.**
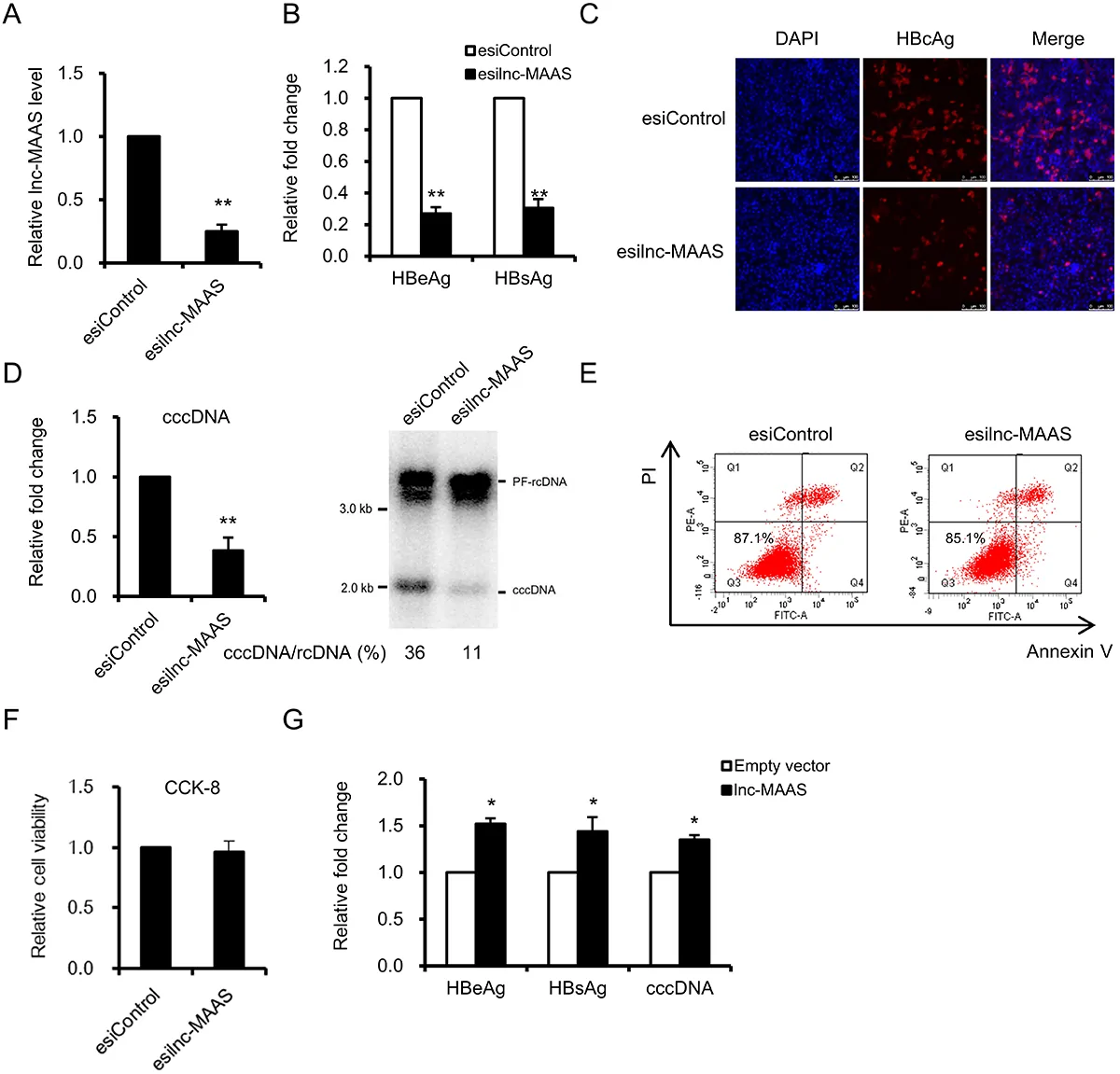
Lnc-MAAS is required for HBV infection in HepG2-hNTCP cells. (A) The knockdown efficiency of lnc-MAAS was confirmed by qPCR. (B) The levels of HBsAg and HBeAg in the supernatants were measured via ELISA. (C) The infected cells were stained with an HBcAg antibody and visualized via fluorescence microscopy. (D) The level of cccDNA in the infected cells was determined by qPCR and Southern blot. (E–F) HepG2-hNTCP cells were transfected with 30 nM esiRNA against lnc-MAAS for 72 h. (E) The effect of lnc-MAAS knockdown on cell apoptosis was analyzed via the Annexin V/PI assay. (F) The effect of lnc-MAAS knockdown on cell viability was determined by performing a CCK-8 assay. (G) Equivalent amounts of HBV virions were used to infect lnc-MAAS-overexpressing cells and control cells. Five d post-infection, the levels of secreted viral antigens and cccDNA in the cells were determined via ELISA and qPCR, respectively. The results are shown as the mean values ± standard deviations. Statistical analysis was performed via Student’s *t*-test (two-tailed). *p < 0.05, **p < 0.01. HBeAg, hepatitis B e antigen; HBsAg, hepatitis B surface antigen; HBcAg, hepatitis B core antigen; cccDNA, covalently closed circular DNA.

Finally, we validated the function of lnc-MAAS in HBV infection using primary human hepatocytes, which are currently considered the gold standard for HBV infection studies *in vitro*. Consistent with the results obtained from HepG2-NTCP cells, silencing of lnc-MAAS in primary human hepatocytes resulted in a dramatic reduction of secreted HBeAg, intracellular HBcAg expression, and intracellular cccDNA levels (Figure 4).

**Figure 4.**
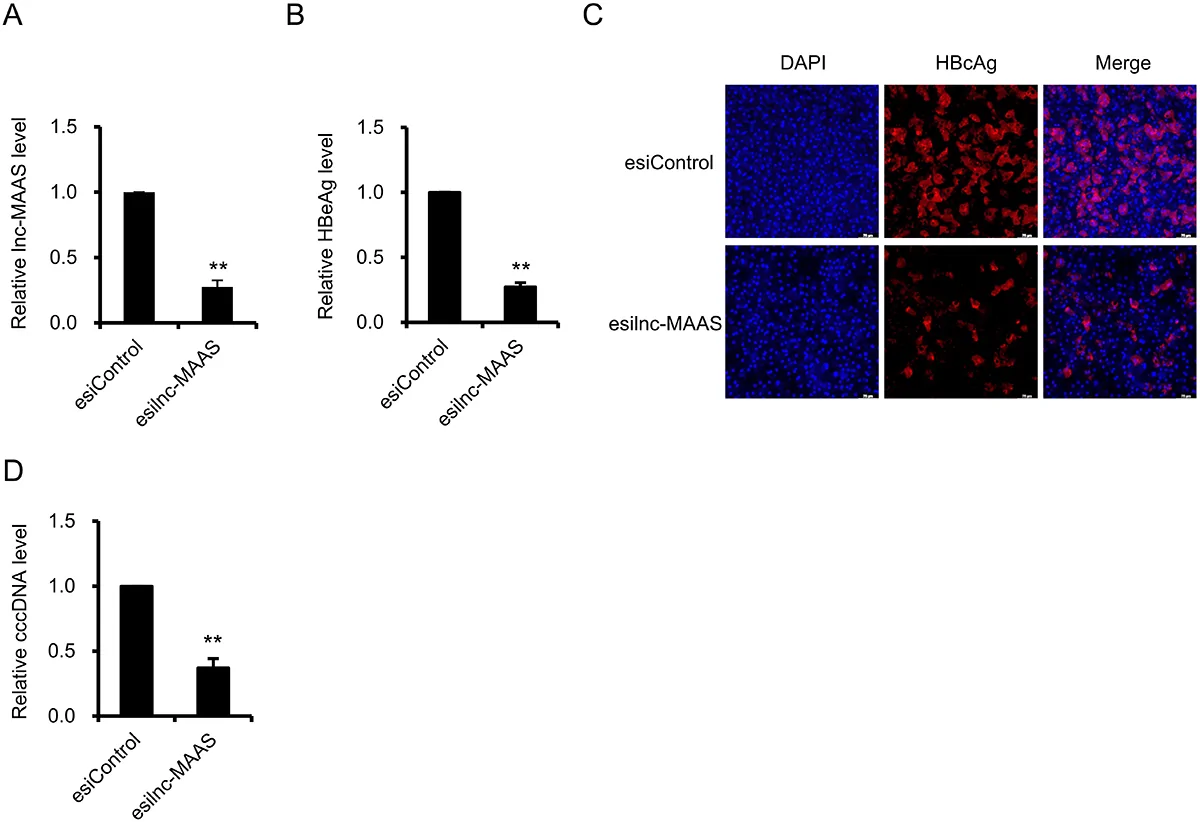
Knockdown of lnc-MAAS inhibits HBV infection in primary human hepatocytes. (A to D) Primary human hepatocytes were transfected with 30 nM esiRNA against lnc-MAAS and then infected with HBV. (A) The knockdown efficiency of lnc-MAAS was confirmed by qPCR. (B) The levels of HBeAg in the supernatants were measured via ELISA. (C) The infected cells were stained with an HBcAg antibody and visualized via fluorescence microscopy. (D) The level of cccDNA in the infected cells was determined by qPCR. The results are shown as the mean values ± standard deviations. Statistical analysis was performed via Student’s *t*-test (two-tailed). *p < 0.05, **p < 0.01. HBeAg, hepatitis B e antigen; HBcAg, hepatitis B core antigen; cccDNA, covalently closed circular DNA.

### Lnc-MAAS is involved in HBV cccDNA formation

We next investigated which step of HBV infection was affected by lnc-MAAS. We employed an esiRNA targeting the HBV receptor NTCP to serve as a positive control. As expected, the downregulation of NTCP reduced viral attachment, whereas HBV attachment was not affected in the lnc-MAAS-knockdown cells (Figure 5(A)). Next, by performing an HBV internalization assay, we found that the depletion of lnc-MAAS did not decrease the level of internalized HBV DNA (Figure 5(B)). We next extracted protein-free rcDNA (PF-rcDNA) from the nuclei of infected cells and found that depletion of lnc-MAAS did not affect the level of PF-rcDNA (Figure 5(C,D)). Thus, our data suggest that lnc-MAAS is involved in either PF-rcDNA to cccDNA conversion or cccDNA turnover. To monitor the impact of lnc-MAAS on cccDNA turnover, we infected HepG2-hNTCP cells with HBV and treated the cells with entecavir (ETV) from 3 d post-infection to inhibit the formation of new rcDNA and the replenishment of cccDNA. On day 5 post-infection, we transfected the cells with lnc-MAAS esiRNA and then analyzed the cccDNA levels on days 3, 5 and 7 post-transfection. Our data revealed that lnc-MAAS had no significant effect on the established HBV cccDNA pool (Figure 5(E)), suggesting that it is not involved in the regulation of cccDNA turnover but is likely required for cccDNA formation.

**Figure 5.**
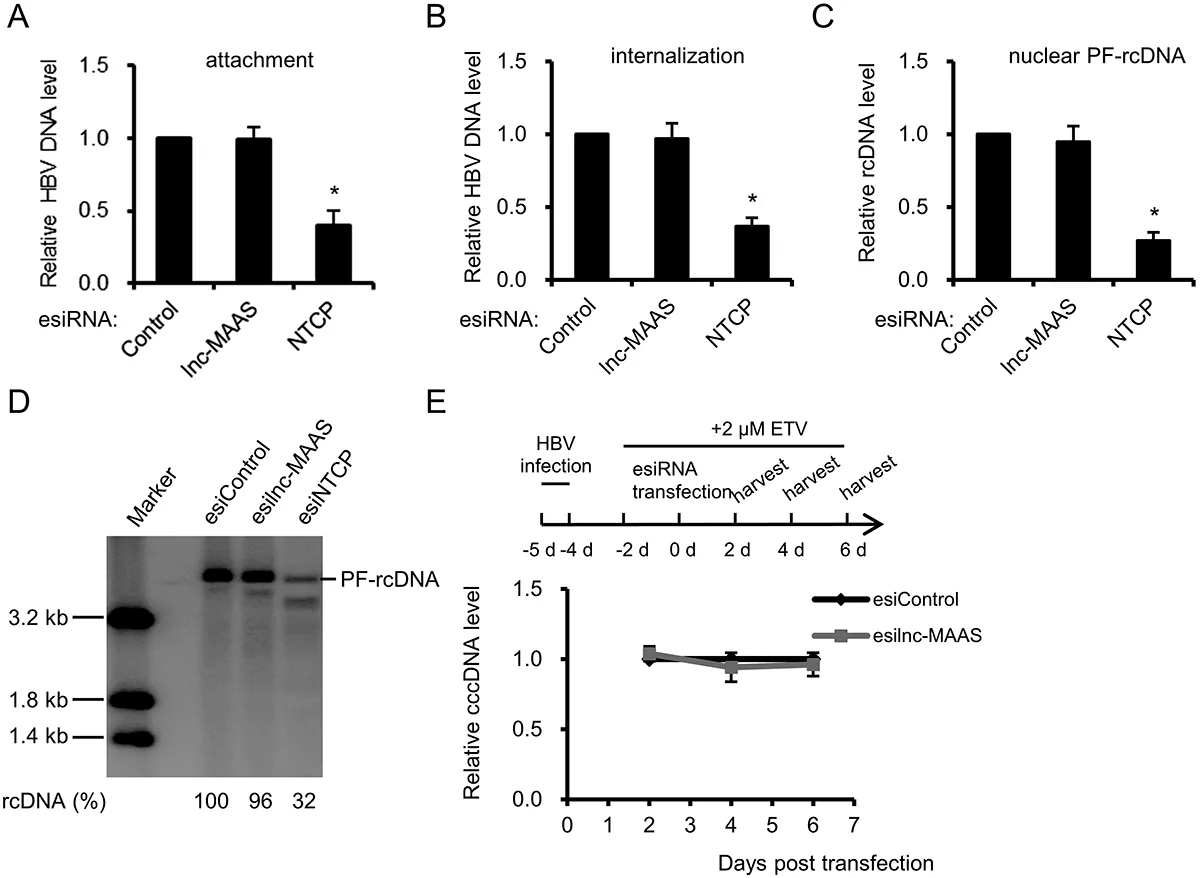
Lnc-MAAS is involved in HBV cccDNA formation. (A to D) HepG2-hNTCP cells were transfected with 30 nM indicated esiRNAs. Two d after transfection, the cells were harvested with trypsin, and the suspended cells were incubated with the HBV inoculum at 4°C for 3 h to allow HBV to attach to the cells. Free HBV was removed by washing the cells with PBS. (A) Total DNA from the washed cells was extracted, and the amount of attached HBV DNA was quantified via qPCR. (B) The washed cells were cultured at 37°C for an additional 16 h. Intracellular HBV DNA was quantified by qPCR. (C-D) The washed cells were cultured at 37°C for an additional 16 h. The nuclei of the cells were isolated, and PF-rcDNA was extracted and quantified via qPCR (C) and Southern blot (D). (E) Effect of lnc-MAAS knockdown on cccDNA stability. The experimental scheme is shown at the top. The level of cccDNA was quantified via qPCR. The results are shown as the mean values ± standard deviations. Statistical analysis was performed via one-way ANOVA with Dunnett’s posttest. *p < 0.05. PF-rcDNA, protein-free relaxed circular DNA; cccDNA, covalently closed circular DNA; ETV, entecavir.

### The function of lnc-MAAS in HBV cccDNA formation is mediated by NABP1

Next, we investigated the mechanism by which lnc-MAAS affects cccDNA formation. Previous studies have shown that lnc-MAAS exerts its biological functions by regulating the expression of protein-coding genes [26–29]. Therefore, we performed RNA sequencing in lnc-MAAS-knockdown HepG2-hNTCP cells to search for gene candidates that are regulated by lnc-MAAS (Supplementary Figure S3A). Given that the formation of cccDNA depends mainly on the host DNA repair machinery, four protein genes, which were previously reported to function in the host DNA repair response, were selected as potential targets (Supplementary Figure S3B). We first verified the expression levels of these candidates via qPCR. Consistent with the RNA sequencing data, NABP1 and CETN2 were downregulated in lnc-MAAS-knockdown cells, whereas RTEL1 and PER1 were upregulated by depletion of lnc-MAAS (Figure 6(A)). With the exception of NABP1, the other three candidate genes presented low expression in HepG2-hNTCP cells. By performing a loss-of-function experiment, we found that only the knockdown of the top candidate NABP1, a single-stranded DNA‑binding protein, significantly reduced the level of cccDNA in the infected cells (Figure 6(B)). The function of NABP1 in cccDNA formation was further verified with two independent siRNAs (Figure 6(C,D)).

**Figure 6.**
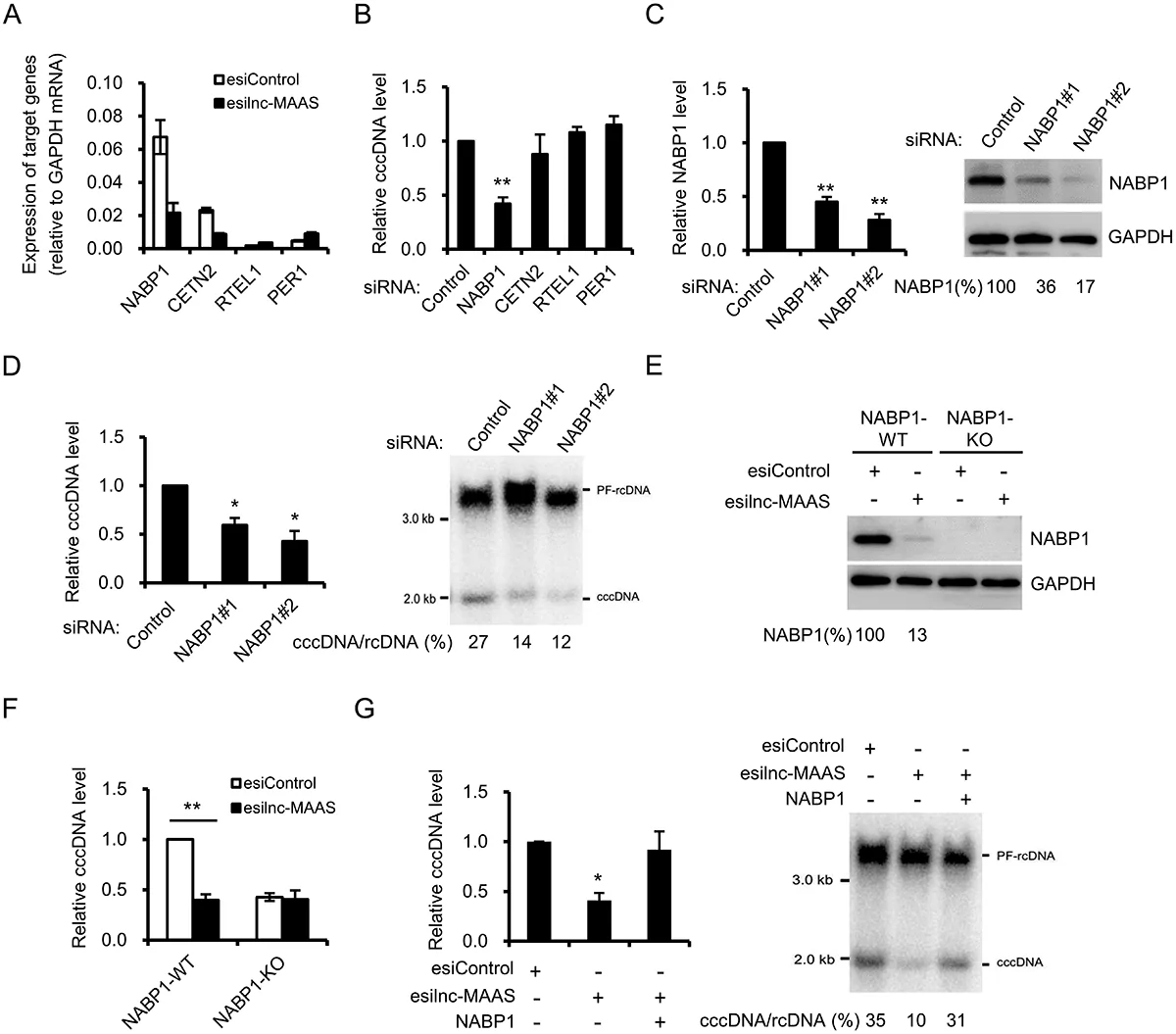
NABP1 mediates the function of lnc-MAAS in HBV cccDNA formation. (A) The expression levels of candidate genes in lnc-MAAS-depleted HepG2-hNTCP cells and control cells were measured by qPCR. (B) HepG2-hNTCP cells were transfected with 30 nM indicated siRNAs and then infected with HBV 2 d after transfection. Five d post-infection, total DNA was extracted, and the cccDNA level was measured via qPCR. (C-D) HepG2-hNTCP cells were transfected with two siRNAs against NABP1 and then infected with HBV 2 d after transfection. (C) The knockdown efficiencies of NABP1 siRNAs were confirmed by measuring NABP1 mRNA levels via qPCR and NABP1 protein levels via immunoblotting. (D) The level of cccDNA was determined by qPCR and Southern blot. (E‑F) HepG2-hNTCP-WT and HepG2-hNTCP-NABP1-KO cells were transfected with 30 nM control esiRNA and lnc-MAAS esiRNA, respectively. Two d post-transfection, the cells were infected with HBV. (E) The expression level of NABP1 was measured via immunoblotting. (F) The level of cccDNA in the infected cells was quantified by qPCR. (G) HepG2-hNTCP cells were transfected with 30 nM indicated esiRNAs and the lentivirus encoding NABP1 was used to transduce the lnc-MAAS-knockdown cells. Two d post‑transfection, the cells were infected with HBV. Five d post-infection, the level of cccDNA in the infected cells was quantified by qPCR and Southern blot. Statistical analysis was performed via two-tailed Student’s *t*-test or one-way ANOVA with Dunnett’s posttest (B-D). *p < 0.05, **p < 0.01. cccDNA, covalently closed circular DNA.

Next, we investigated whether the function of lnc-MAAS in cccDNA formation is mediated by NABP1. To this end, we established a stable NABP1-knockout HepG2-hNTCP cell line (Supplementary Figure S4) and then measured the effect of lnc-MAAS on cccDNA formation in the NABP1-knockout cells. We found that knockout of NABP1 almost entirely abolished the effect of lnc-MAAS on HBV cccDNA formation (Figure 6(E,F)). Furthermore, we found that restoration of NABP1 expression in lnc-MAAS-knockdown HepG2-hNTCP cells can rescue the formation of HBV cccDNA (Figure 6(G)). To further validate the generality of this regulatory axis in a cell model supporting HBV replication, we repeated the above experiments in HepAD38 cells and obtained results consistent with those in HepG2‑hNTCP cells (Supplementary Figure S5). Taken together, these data suggest that NABP1 is responsible for the function of lnc-MAAS in HBV cccDNA formation.

### Lnc-MAAS transcriptionally regulates the expression of NABP1

We next investigated the effect of lnc-MAAS on the expression of NABP1. We measured the expression level of NABP1 in lnc-MAAS-knockdown and -overexpressing cells via both qPCR and immunoblotting. We found that depletion of lnc-MAAS decreased the level of NABP1 (Figure 7(A)), whereas the expression of NABP1 was increased by overexpression of lnc-MAAS (Figure 7(B)), suggesting that lnc-MAAS promotes the expression of NABP1. We next asked how lnc-MAAS modulates the expression of NABP1. To answer this question, we investigated the impact of lnc-MAAS on the transcriptional activity of the *NABP1* promoter and on the stability of *NABP1* mRNA. We cloned the *NABP1* promoter into a luciferase reporter vector and then transfected this reporter plasmid into lnc-MAAS-knockdown and -overexpressing cells. Our data revealed that knockdown of lnc-MAAS significantly reduced luciferase activity in cells, whereas overexpression of lnc-MAAS increased luciferase activity (Figure 7(C)). To determine the effect of lnc-MAAS on *NABP1* mRNA stability, we used actinomycin D to block the transcription and measured the decay kinetics of *NABP1* mRNA in lnc-MAAS-knockdown and control cells. We found that lnc-MAAS had no significant effect on the turnover of *NABP1* mRNA (Figure 7(D)). Thus, our data suggest that lnc-MAAS modulates the expression of NABP1 by regulating the transcriptional activity of the *NABP1* promoter.

**Figure 7.**
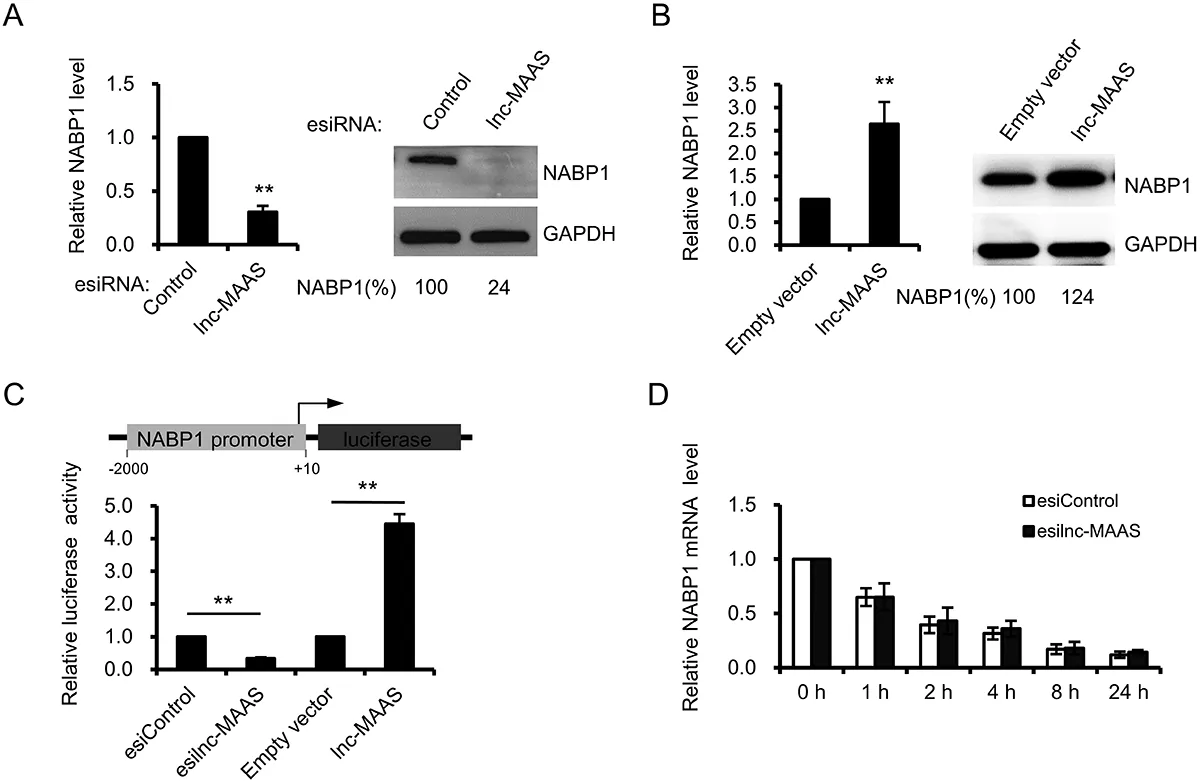
Lnc-MAAS regulates the expression of NABP1. (A) The expression levels of NABP1 in lnc-MAAS-knockdown HepG2-hNTCP cells and control cells were measured by qPCR and immunoblotting. (B) The expression levels of NABP1 in lnc-MAAS-overexpressing HepG2-hNTCP cells and control cells were measured by qPCR and immunoblotting. (C) NABP1 promoter reporter vectors were cotransfected with lnc-MAAS esiRNA or lnc-MAAS-expressing plasmids into HepG2 cells. The activity of luciferase in the cells was determined via a luciferase reporter assay 3 d after transfection. (D) HepG2-hNTCP cells were transfected with 30 nM indicated esiRNAs and then treated with actinomycin D (5 µg/mL) 1 day after transfection for various time periods as indicated. The expression level of NABP1 was measured by qPCR. The NABP1 mRNA levels in both Lnc-MAAS knockdown and control cells harvested at 0 h were set as 1. The results are shown as the mean values ± standard deviations. Statistical analysis was performed via Student’s *t*-test (two-tailed). *p < 0.05.

## Discussion

In the present study, to identify novel host lncRNAs involved in HBV infection, we conducted a loss-of-function screen via an esiRNA library targeting human lncRNAs. EsiRNAs are generated by using bacterial RNase III or recombinant Dicer to enzymatically digest long double-stranded RNA (dsRNA), which is produced by *in vitro* transcription using the PCR product of the desired target transcript region as a template [17,30]. Compared with chemically synthesized short interfering RNAs (siRNAs), esiRNAs have been shown to possess greater silencing capacity and fewer off-target effects for the knockdown of lncRNAs, since each esiRNA is a pool of several hundred individual siRNAs [30,31]. Owing to the advantages of esiRNA technology, the lncRNA esiRNA library is a useful tool for investigating the functions of cellular lncRNAs [32,33]. However, this library has one major drawback. Because of the massive amount of sequencing data generated over the last decade, a number of novel lncRNAs have been identified since the esiRNA library used here was constructed. These lncRNAs were not included in our screen, and their roles in HBV infection remain unclear.

Through loss-of-function screening, we identified 19 host lncRNAs that are associated with HBV infection. Interestingly, our data revealed that 10 of the 19 lncRNAs affected HBV cccDNA levels in infected cells. These lncRNAs differ from previously reported lncRNAs, such as DLEU2, HOTTIP and LINC01431, which all exert their functions by modulating cccDNA transcription but have no effect on cccDNA formation [14–16]. In our screen, we used NTCP-overexpressing HepG2 cells, a robust, convenient, less expensive *in vitro* model for studying HBV infection [34]. In this model, HBV cccDNA is generated from the rcDNA derived from incoming virions [35]. Thus, host lncRNAs acting on any early events of the HBV life cycle, from viral entry to rcDNA repair in the nucleus, could lead to altered cccDNA levels. To date, increasing evidence shows that the functions of cellular lncRNAs are highly heterogeneous. For example, they have been reported to function in endocytosis, cytoplasmic to nuclear trafficking, and cellular DNA repair [36–39]. Hence, the identified lncRNAs could affect the HBV cccDNA level by being involved in these cellular processes.

We further functionally characterized one candidate lncRNA named lnc-MAAS. Our data showed that the expression level of lnc-MAAS was increased by HBV infection, and depletion of lnc-MAAS significantly reduced cccDNA levels in infected cells, suggesting that lnc-MAAS is a proviral factor of HBV. A previous study showed that the overexpression of lnc-MAAS promotes cell proliferation in HBV-related HCC [24]. Consistently, we found that the knockdown of lnc-MAAS slightly reduced the growth rate of HepG2-hNTCP cells (data not shown). However, this biological role of lnc-MAAS cannot explain the decreased cccDNA formation in lnc-MAAS-depleted cells, since slower cell proliferation is supposed to decelerate cccDNA loss during cell division, which should result in increased cccDNA levels [40].

Further study revealed that lnc-MAAS participates in cccDNA formation by modulating the expression of NABP1. Although lncRNAs often act on multiple molecular targets, our experimental pipeline suggests that NABP1 is the specific downstream mediator for the cccDNA-regulating function of lnc-MAAS. This was confirmed by the screening of DNA damage response-related candidates, where only NABP1 knockdown mimicked the effect of lnc-MAAS depletion on cccDNA, and by the genetic rescue experiments demonstrating that the effect of lnc-MAAS is mainly dependent on the presence of NABP1. Previous studies showed that lnc-MAAS can regulate gene expression at the transcriptional level [29] or the post-transcriptional level by acting as a sponge of miRNAs [26–28]. Our results from the promoter-reporter assay indicate that lnc-MAAS regulates NABP1 expression at the transcriptional level. However, the detailed molecular mechanism remains to be investigated. In addition, although we did not find any liver-expressed miRNAs that can target both lnc-MAAS and NABP1 mRNAs by using prediction tools, we cannot exclude the possibility that lnc-MAAS could also increase the expression of NABP1 by sponging certain miRNAs.

NABP1 is also called human single-stranded DNA‑binding protein 2 (hSSB2). Single-stranded DNA‑binding proteins (SSBs) play critical roles in DNA replication, recombination, DNA damage detection, and repair [41]. As a member of the SSB family, NABP1 has been proven to participate in the cellular DNA damage response to maintain genomic stability [42–44]. Notably, our results showed that NABP1 reconstitution in lnc-MAAS-knockdown cells led to a marked increase in cccDNA levels, while the PF-rcDNA reservoir remained largely stable or showed a slight decreasing trend (Figure 6(G)). This observation suggests that NABP1 enhances the net efficiency of converting protein-free relaxed circular DNA (PF-rcDNA) into covalently closed circular DNA (cccDNA). Given NABP1’s known role in DNA damage response and single-stranded DNA binding [41,44], it is plausible that NABP1 facilitates the final repair steps of the rcDNA‑to‑cccDNA conversion process. Recently, Verrier et al. performed a loss-of-function screen targeting 239 genes encoding the human DNA damage response machinery and identified NABP1 as one of the candidate host factors required for the intracellular cccDNA amplification pathway [45]. However, their studies focused only on the top candidate named YBX1. Therefore, the function of NABP1 in cccDNA formation has yet to be fully proven, and the detailed mechanism involved remains to be elucidated [45]. These data, combined with our results, suggest that NABP1 is essential for both *de novo* cccDNA formation and intracellular cccDNA amplification. Interestingly, a previous study showed that NABP1 knockout mice have no apparent defects [46], suggesting that the biological functions of NABP1 in vivo are dispensable. Thus, NABP1 could be a promising target for treating HBV infection.

Emerging evidence has revealed that noncoding RNAs are key modulators of complex regulatory networks in human diseases, operating through diverse molecular mechanisms [47,48]. In this context, lnc‑MAAS may intersect with host immune pathways beyond its role in cccDNA formation. A recent bioinformatics study in hepatocellular carcinoma (HCC) revealed that lnc‑MAAS is enriched in immune‑related pathways [49], and functional evidence from non‑liver contexts supports a role for lnc-MAAS in modulating inflammatory responses [50,51]. More directly relevant to HBV infection, the viral antigen HBeAg has been demonstrated to upregulate lnc‑MAAS in M2 macrophages by enhancing its m^6^A modification [24]. Given that M2‑polarized tumor‑associated macrophages contribute to immune impairment in chronic HBV infection, these findings raise the intriguing possibility that lnc‑MAAS may not only facilitate HBV cccDNA formation but also participate in shaping the antiviral immune microenvironment. This notion is conceptually consistent with the broader principle that antigen exposure and immune activation can trigger dysregulated inflammatory responses leading to tissue pathology, as exemplified by immune‑mediated adverse conditions following vaccination in other clinical settings [52,53]. Nevertheless, the precise role of lnc‑MAAS in HBV‑related immune regulation remains to be defined, and future investigations are warranted to explore this possibility.

Beyond its role in host immune-related pathway, lnc‑MAAS may also play a role in host metabolic pathways. Bioinformatics analyses have revealed that lnc‑MAAS is also enriched in metabolism‑related pathways in hepatocellular carcinoma (HCC) [49], and its dysregulation has been associated with altered amino‑acid and lipid metabolism in colon cancer‑derived liver metastasis [54]. It has been shown that host metabolic reprogramming frequently contributes to disease progression [55,56]. Future investigations focusing on the interplay between this lnc-MAAS/NABP1 regulatory axis and cellular metabolic pathways will be helpful to fully understand its biological significance in HBV-related liver diseases.

In summary, via a loss-of-function approach, we report the identification of lnc-MAAS as a novel host lncRNA required for HBV infection. Moreover, our data suggest that lnc-MAAS participates in HBV cccDNA formation by regulating the expression of NABP1. While immediate clinical translation remains a major challenge, our results provide a crucial mechanistic framework for future mechanism-guided intervention strategies. As emphasized in recent developments in drug discovery, a thorough understanding of host-virus molecular interactions is the prerequisite for designing precise antiviral therapies [57,58]. Therefore, the established lnc-MAAS/NABP1 regulatory axis represents a promising candidate for host-directed antiviral research and warrants further validation in appropriate translational models.

## Supplementary Material

Supplementary Figure legends.docx

Supplementary Figure 5.JPG

Supplementary Figure 3.JPG

Supplementary Table 1.docx

Supplementary Figure 2.JPG

Supplementary Table 2.docx

Supplementary Figure 1.JPG

Supplementary Figure 4.JPG

## Data Availability

The data that support the findings of this study are openly available in Zenodo at https://doi.org/10.5281/zenodo.18596594 [59].
