# Supplementary material for: The long noncoding RNA MAPKAPK5-AS1 promotes the formation of hepatitis B virus cccDNA by modulating the expression of NABP1
Source: Virulence. 2026 Oct 12;17(1):2745300. doi: 10.1080/21505594.2026.2745300 (PMC13671438; doi:10.1080/21505594.2026.2745300)
Supplement: Supplementary file 1 [file KVIR_A_2745300_SM7011.docx]

**Supplementary Table 2**. List of lncRNAs identified in the primary screen

| Number | LNCipedia  Gene ID | Relative HBeAg  Level | Expression in HepG2 cells (FPKM) | |
| --- | --- | --- | --- | --- |
|  |  |  | cytosol | nucleus |
| 1 | LINC01762 | 0.259 ± 0.044 | 0.189868 | 2.839058 |
| 2 | MAPKAPK5-AS1 | 0.270 ± 0.022 | 7.348139 | 7.136052 |
| 3 | lnc-IDH3B-1 | 0.288 ± 0.074 | 0.085903 | 0.272592 |
| 4 | lnc-COL6A3-14 | 0.333 ± 0.047 | 1.570876 | 1.234848 |
| 5 | lnc-PIF1-1 | 0.333 ± 0.085 | 0.230909 | 0.298623 |
| 6 | lnc-ATP6V1G3-4 | 0.348 ± 0.093 | 0.402906 | 0.387190 |
| 7 | lnc-TMCC1-12 | 0.353 ± 0.069 | 0.074602 | 0.394550 |
| 8 | lnc-FBXW4-1 | 0.361 ± 0.053 | 0.000000 | 0.012692 |
| 9 | LINC02367 | 0.364 ± 0.063 | 0.127645 | 1.104034 |
| 10 | lnc-CENPP-1 | 0.374 ± 0.031 | 0.211907 | 0.085629 |
| 11 | lnc-KIAA1257-1 | 0.418 ± 0.092 | 0.146535 | 0.581244 |
| 12 | lnc-MTA3-12 | 0.422 ± 0.030 | 0.105275 | 0.159587 |
| 13 | lnc-FEZ2-7 | 0.449 ± 0.048 | 2.001655 | 0.904870 |
| 14 | lnc-KCNE1B-3 | 1.960 ± 0.302 | 4853.813 | 4232.610 |
| 15 | lnc-NTN3-1 | 1.990 ± 0.165 | 0.183395 | 0.436471 |
| 16 | lnc-USP53-6 | 1.990 ± 0.107 | 0.339471 | 0.678484 |
| 17 | lnc-RIMKLB-1 | 2.160 ± 0.152 | 0.011344 | 0.229485 |
| 18 | ARHGAP5-AS1 | 2.176 ± 0.176 | 0.187817 | 0.126758 |
| 19 | lnc-TMPPE-2 | 2.343 ± 0.302 | 0.269057 | 2.232980 |
