# Supplementary material for: The long noncoding RNA MAPKAPK5-AS1 promotes the formation of hepatitis B virus cccDNA by modulating the expression of NABP1
Source: Virulence. 2026 Oct 12;17(1):2745300. doi: 10.1080/21505594.2026.2745300 (PMC13671438; doi:10.1080/21505594.2026.2745300)
Supplement: Supplementary file 2 [file KVIR_A_2745300_SM7013.docx]

**Supplementary Table 1**. Sequences of primers and siRNAs used in this study

| **qPCR primers:** | | |
| --- | --- | --- |
| lnc-MAAS | Sense | GAGATTCACGCCTGGAAAAC |
|  | Anti-sense | GACGAAAGGAGGAAATGCAG |
| hNTCP | Sense | AAGGACAAGGTGCCCTATAAAGG |
|  | Anti-sense | TTGAGGACGATCCCTATGGTG |
| cccDNA | Sense | TGCACTTCGCTTCACCT |
|  | Anti-sense | AGGGGCATTTGGTGGTC |
| NABP1 | Sense | CAGTGAACCCAACCCAGATTATC |
|  | Anti-sense | GGTCCAAATGTACCTGTACCCAT |
| CETN2 | Sense | GCATCAAGTTCTCAGCGAAAAAG |
|  | Anti-sense | CCATCCGCATCGAAAAGATCAA |
| RTEL1 | Sense | CCTGAATGGTGTGACCGTAGA |
|  | Anti-sense | CCTGTACCCGTAGGGCTCTC |
| PER1 | Sense | AGTCCGTCTTCTGCCGTATCA |
|  | Anti-sense | AGCTTCGTAACCCGAATGGAT |
| HBV 3.5kb transcripts | Sense | GAGTGTGGATTCGCACTCC |
|  | Anti-sense | GAGGCGAGGGAGTTCTTCT |
| Total HBV transcripts | Sense | TCACCAGCACCATGCAAC |
|  | Anti-sense | AAGCCACCCAAGGCACAG |
| GAPDH | Sense | CTCTGCTCCTCCTGTTCGAC |
|  | Anti-sense | TTAAAAGCAGCCCTGGTGAC |
| Pre-GAPDH | Sense | CCACCAACTGCTTAGCACC |
|  | Anti-sense | CTCCCCACCTTGAAAGGAAAT |
| **Cloning primers:** | | |
| NABP1 promoter | Sense | TACGCGTGCTAGCCCGGGCTCGAGGCCTGAACAGCCCAACCTAA |
|  | Anti-sense | CAACAGTACCGGAATGCCAAGCTTAAACAGCGACTGATGAACACG |
| **siRNA target sequences:** | | |
| lnc-MAAS-siRNA |  | GCATTAACCTTACGTGGAGTT |
|  |  |  |
| NABP1-siRNA #1 |  | GATGAGATCGGAGGTCTTATA |
|  |  |  |
| NABP1-siRNA #2 |  | GAAAGGATGTCTGACACTTTA |
|  |  |  |
| CETN2-siRNA |  | CTGGGCTTTGAACCCAAGAAA |
|  |  |  |
| RTEL1-siRNA |  | CCGTACAATTACTTGTTGGAT |
|  |  |  |
| PER1-siRNA |  | CCAGCACCACTAAGCGTAAAT |
|  |  |  |
