# Supplementary material for: The long noncoding RNA MAPKAPK5-AS1 promotes the formation of hepatitis B virus cccDNA by modulating the expression of NABP1
Source: Virulence. 2026 Oct 12;17(1):2745300. doi: 10.1080/21505594.2026.2745300 (PMC13671438; doi:10.1080/21505594.2026.2745300)
Supplement: Supplementary file 3 [file KVIR_A_2745300_SM7016.docx]

**Supplementary Figure 1. HBV infection is inhibited by siRNA-mediated knockdown of lnc-MAAS.** (A) The knockdown efficiency of lnc-MAAS was confirmed by qPCR. (B) The levels of HBsAg and HBeAg in the supernatants were measured via ELISA. (C) The infected cells were stained with an HBcAg antibody and visualized by fluorescence microscopy. (D) The level of cccDNA in the infected cells was determined by qPCR and Southern blot. The results are shown as the mean values ± standard deviations. Statistical analysis was performed via Student's *t*-test (two-tailed). **P* < 0.05. HBeAg, hepatitis B e antigen; HBsAg, hepatitis B surface antigen; HBcAg, hepatitis B core antigen; cccDNA, covalently closed circular DNA.

**Supplementary Figure 2. Effect of lnc‑MAAS knockdown on HBV infection across different genotypes.** HepG2‑hNTCP cells were transfected with 30 nM control esiRNA or lnc‑MAAS esiRNA for 48 h and then infected with HBV genotypes A, B, C, or D. Five days post‑infection, the levels of HBeAg in the culture supernatants (A) and intracellular cccDNA (B) were measured by ELISA and qPCR, respectively. The results are shown as the mean values ± standard deviations. Statistical analysis was performed via Student's *t*-test (two‑tailed).  ***P* < 0.01. HBeAg, hepatitis B e antigen; cccDNA, covalently closed circular DNA; gtA, genotype A; gtB, genotype B; gtC, genotype C; gtD, genotype D.

**Supplementary Figure 3. Identification of differentially expressed genes in lnc-MAAS depleted cells.** (A) The volcano map of the differentially expressed genes in lnc-MAAS depleted cells. HepG2-hNTCP cells were transfected with control esiRNA or lnc-MAAS esiRNA. Two days posttransfection, the total RNAs in control cells and lnc-MAAS depleted cells were extracted and analyzed by RNA sequencing. (B) Among the differentially expressed genes, four genes involved in the host DNA repairing response were selected.

**Supplementary Figure 4. Establishment of NABP1 knock-out cell lines.** (A) Representative image of sanger sequencing of wild type and NABP1 knock-out cells. (B) The levels of NABP1 in wild-type and NABP1 knock-out cells were measured via immunoblotting. (C) The cell viability of wild-type and NABP1 knock-out cells was determined via performing a CCK-8 assay. The results are shown as the mean values ± standard deviations. WT, wild-type; KO, knock-out.

**Supplementary Figure 5. Validation of the lnc‑MAAS/NABP1 regulatory axis in HepAD38 cells.** HepAD38 cells cultured in the absence of tetracycline were transfected with 30 nM indicated esiRNAs and the lentivirus encoding NABP1 was used to transduce the lnc-MAAS-knockdown cells. Seven days posttransfection, the level of cccDNA in the cells was quantified by qPCR. The results are shown as the mean values ± standard deviations. Statistical analysis was performed using one‑way ANOVA with Dunnett's post‑test.  *P < 0.05.
